# Cholesterol Lowering Modulates T Cell Function *In Vivo* and *In Vitro*

**DOI:** 10.1371/journal.pone.0092095

**Published:** 2014-03-19

**Authors:** Kuang-Yuh Chyu, Wai Man Lio, Paul C. Dimayuga, Jianchang Zhou, Xiaoning Zhao, Juliana Yano, Portia Trinidad, Tomoyuki Honjo, Bojan Cercek, Prediman K. Shah

**Affiliations:** Oppenheimer Atherosclerosis Research Center, Division of Cardiology, Cedars-Sinai Heart Institute, Cedars-Sinai Medical Center, Los Angeles, California, United States of America; Maastricht University, Netherlands

## Abstract

**Background:**

The lipid milleu exacerbates the inflammatory response in atherosclerosis but its effect on T cell mediated immune response has not been fully elucidated. We hypothesized that lipid lowering would modulate T cell mediated immune function.

**Methods and Results:**

T cells isolated from human PBMC or splenic T cells from apoE-/- mouse had higher proliferative response to T cell receptor (TCR) ligation in medium supplemented with 10% fetal bovine serum (FBS) compared to medium with 10% delipidated FBS. The differences in proliferation were associated with changes in lipid rafts, cellular cholesterol content, IL-10 secretion and subsequent activation of signaling molecule activated by TCR ligation. Immune biomarkers were also assessed in vivo using male apoE-/- mice fed atherogenic diet (AD) starting at 7 weeks of age. At 25 weeks of age, a sub-group was switched to normal diet (ND) whereas the rest remained on AD until euthanasia at 29 weeks of age. Dietary change resulted in a lower circulating level of cholesterol, reduced plaque size and inflammatory phenotype of plaques. These changes were associated with reduced intracellular IL-10 and IL-12 expression in CD4^+^ and CD8^+^ T cells.

**Conclusion:**

Our results show that lipid lowering reduces T cell proliferation and function, supporting the notion that lipid lowering modulates T cell function *in vivo* and *in vitro*.

## Introduction

Pharmacological lipid lowering using statins is the primary medical therapy to reduce morbidity and mortality from atherosclerotic cardiovascular disease. This beneficial effect has been attributed to the plaque-stabilizing effects of cholesterol lowering accompanied by reduced inflammatory phenotype of atherosclerotic plaques as demonstrated both in clinical [1]–[3] and preclinical studies [4], [5]. However, it is not known if the reduced inflammatory response is due to the direct effect of cholesterol lowering as demonstrated in preclinical studies or due to pleiotropic effects of statin [6]–[9].

Cellular cholesterol plays a significant role in T cell responses. Increased accumulation of intracellular cholesterol content polarises T cells toward a more inflammatory phenotype [10], [11]; whereas hypercholesterolemic milieu alters T helper response [12]–[15]. It is known that cholesterol lowering by medication favorably affects the inflammatory response, but whether it affects T cell response remains unclear. Dietary modification to lower circulating cholesterol level is another effective strategy to modify atherosclerotic cardiovascular diseases. This benefit has been primarily attributed to its cholesterol lowering effect; however whether such cholesterol lowering affects T cell function also remains unknown. Previous studies have primarily studied the T cell response in lipid loading condition comparing mice on high cholesterol chow vs. mice on normal chow [12], [15]. Change of T cell function has not been reported with cholesterol lowering after a period of hypercholesterolemia, a scenario similar to what occurs in clinical practice. Hence we conducted a series of *in vitro* and *in vivo* experiments to test the hypothesis that cholesterol lowering favorably modulates T cell function. Cholesterol lowering was achieved with dietary modification without the use of pharmacologic agents to avoid the confounding effects of these agents on immune function.

## Materials and Methods

### Lipid lowering by diet in mice

Male apoE-/- mice on C57BL/6 background were purchased from Jackson Laboratories (Bar Harbor, Me) at 6 weeks of age and were housed in a pathogen-free animal facility accredited by the Association for the Assessment and Accreditation of Laboratory Animal Care International and kept on a 12-hour day/night cycle with unrestricted access to water and food. All mice were fed atherogenic diet (TD88137, Harlan Teklad) starting at the age of 7 weeks. At the age of 25 weeks, one group of mice was switched to normal chow diet (ND mice) and another group remained on atherogenic diet (AD mice). All mice were euthanized at 29 weeks of age. Mouse blood was collected via retro-orbital bleeding under anesthesia by isoflurane. After blood collection, mice were euthanized with overdose of isoflurane. Splenocytes were harvested for flow cytometric analysis and then 200 U of heparin sodium (APP Pharmaceuticals) was injected into the left ventricle before the whole body was perfused with 0.9% saline for 5 minutes at physiological flow rate. Heart base were harvested and embedded in OCT compound (Tissue-Tek, Allegiance) and frozen at −80°C.

### Histomorphometry

Sections from heart bases were stained with Oil-Red-O for plaque sizes, lipid content or MOMA-2 (Serotec) for macrophage immunoreactivity in the aortic sinus using standard protocol and measurements were done by computer-assisted morphometric analysis as previously described [16].

T cells in aortic sinus were stained with anti-mouse CD3 antibody (eBioscience). Frozen sections were fixed in acetone for 5 min at −20°C, blocked with 3% Hydrogen Peroxide with 0.1% sodium azide for 5 min at room temperature and Serum-Free Protein Block (Dako) for 30 min at room temperature and then incubated with 1∶50 w/v CD3 antibody in 5% goat serum at 4°C overnight. On the second day, sections were incubated with 1∶1200 w/v Biotin conjugated goat anti-Rat IgG (Santa Cruz Biotechnology) for 1.5 hour at room temperature and then with Streptavidin-HRP (Dako) for 25 min at room temperature. Positive signals were visualized by AEC Substrate Chromagen (Dako) and hematoxylin was used as counterstain.

### Total and free serum cholesterol

Serum levels of total or free cholesterol were measured using commercially available kits (Wako). Experiments were done following the manufacturer's instruction manuals.

### Flow cytometry

Suspensions of homogenized spleen cells were pelleted and resuspended in RBC lysis buffer (8.26 g/L NH4Cl, 1 g/L KHCO3 and 0.037 g/L EDTA). After 5 min of lysis at room temperature, cells were washed twice with FACS buffer (1XPBS, 1% BSA and 0.1% sodium azide). To assess the intracellular IFN-γ, IL-10 and IL-12 levels in T cells, cells were incubated with 3 μg/ml Brefeldin A (eBioscience) for 4 hours first to block the secretion of intracellular proteins. Cells were then stained for surface antigens with anti-mouse FITC-CD4 and PE-CD8b antibodies (BD Pharmingen) and then fixed and permeabilized with Fixation/Permeabilization Buffer (eBioscience) for one hour at 4°C. Staining of intracellular cytokines with anti-mouse APC-IFN-γ, APC-IL-10 and PerCP-Cy5.5-IL-12 antibodies (eBioscience) was carried out in 1X Permeabilization Buffer (eBioscience). Samples were analyzed with a LSRII flow cytometer (BD Biosciences) and analyzed using Summit v4.3 software (Dako). A positive signal is defined based on the reading of the corresponding isotype control.

### In vitro T cell proliferation assay

T cells were isolated from the splenocytes of male apoE-/- mice (age of 8 weeks to 19 weeks) using Dynabeads Untouched Mouse T cells kit (Invitrogen). Isolated T cells were loaded with 2.5 μM CFSE (Invitrogen) for 10 min and then seeded at 0.5×10^6^ cells in 1 ml RPMI 1640 medium (invitrogen) supplemented with 10% FBS (Omega Scientific) or 10% delipidated FBS (Cocalico Biologicals Inc.), 1X Antibiotic-Antimycotic (Gibco) and 50 μM β-ME in 12 well plates. All in vitro experiments were performed in medium identical to that just described unless stated otherwise. Anti-mouse CD3/CD28 beads (Invitrogen) were added in a bead-to-cell ratio of 1∶1. Fetal bovine serum contains approximately 20 mg/dl total cholesterol whereas the level of total cholesterol in delipidated FBS was below the detection limit of the cholesterol kit (WACO). Fresh 10% FBS medium or 10% delipidated FBS medium (500 μl) was supplemented to the cells on the 3rd day. On the 5th day, activation beads were removed by magnet and then the cells were stained for CD4 and CD8 as described above using anti-mouse PE-CD4 or PE-CD8b (eBioscience). Additional experiments were also conducted on wild type mice (age of 16 weeks to 19 weeks) and apoE-/- mice (age of 17 weeks to 20 weeks) on the C57BL/6 background using FBS and delipidated FBS of the same lot (Gemini Bio-Products) to avoid the potential batch-to-batch differences in the biochemical contents in the FBS which may affect the experimental outcomes.

The same experiment was performed using human peripheral blood mononuclear cells (AllCells, Emeryville, CA). T cells were isolated from human peripheral mononuclear cells using Dynabeads Untouched Human T cells kit (Invitrogen), activated with anti-human CD3/CD28 beads (Invitrogen) in a bead-to-cell ratio of 1∶2 and stained with anti-human APC-eFluor 780-CD4 and PE-CD8a (eBioscience) as described above. Bead-to-cell ratio of 1∶2 was used as determined by prior optimization experiments.

### Cholesterol and membrane lipid raft determination *in vitro*

Isolated mouse T cells were obtained as described above. After 24 hours incubation in 10% FBS medium or 10% delipidated FBS medium with CD3/CD28 activation beads, cells were harvested to stain for lipid raft with Alexa Fluor 488 Cholera Toxin Subunit B (Invitrogen) on CD4^+^ or CD8^+^ T cells. Samples were always kept on ice or at 4°C to prevent internalization of the lipid raft. Cells were fixed with 2% paraformaldehyde for one hour at 4°C and then stained with anti-mouse PE-CD4 and PerCP-eFluor 710-CD8b (eBioscience) for 30 minutes at 4°C, followed by staining with 100 μl of 8 μg/ml Alexa Fluor 488 Cholera Toxin Subunit B for 15 min at 4°C. Some T cells were stained for Filipin (Sigma) to assess intracellular and surface unesterified cholesterol [17] in CD4^+^ or CD8^+^ T cells. Cells were fixed with 3% paraformaldehyde for one hour at room temperature and then stained with anti-mouse APC-eFluor 780-CD4 and APC-CD8b (eBioscience) for 30 minutes at 4°C, followed by staining with 1 ml of 0.05 μg/ul Filipin for two hour at room temperature.

### Culture medium ELISA

Culture medium was collected at harvest after mouse T cells were incubated with CD3/CD28 activation beads for 4 days from the experiments described above to detect levels of IL-10, IL-12 and IFN-γ by ELISA using standard protocol on Maxisorp 96 well plates. Standards, purified and biotinylated antibody pairs were purchased from eBiosciences. Culture medium was undiluted and signals were produced by avidin-HRP (eBiosciences) and ABTS substrate (SouthernBiotech) and detected at 405 nm.

### Western blot analysis of phosphorylated Zap70

Isolated mouse T cells were obtained as described above. One million cells were resuspended in 30 μl of 10% FBS medium or 10% delipidated FBS medium with CD3/CD28 activation beads in a bead-to-cell ratio of 1∶1. After 2 min of incubation, cells were mixed by pipetting up and down 5 times in RIPA buffer (Sigma-Aldrich) containing complete, Mini protease inhibitor cocktail tablet (Roche Diagnostics) Proteins were extracted by freeze and thaw three times and then centrifuged at 13793 g for 20 min at 4°C. Protein concentrations were determined by Pierce BCA Protein Assay Kit (Thermo Scientific). Ten μg of protein were loaded on a 12% SDS-Page gel and then transferred to nitrocellulose membrane. The membrane was blocked with 5% BSA and then probed with anti phospho-Zap-70, anti Zap-70 or anti α-tubulin (Cell Signaling) as primary antibody and ECL HRP labelled anti rabbit IgG (GE Healthcare) as secondary antibody. Amersham ECL Prime detection reagent (GE Healthcare) was used to visualize protein bands.

### Statistical analysis

Data are presented as mean ± Std. Number of animals in each group is listed in text or figure legend. Numerical data were analyzed by unpaired or paired t-test using GraphPad Prism 3 statistical software when appropriate. P<0.05 was considered as statistically significant.

### Ethics Statement

Mice were housed in a pathogen-free facility at a 12 hours day/night cycle and had unlimited access to food and water. The Cedars-Sinai Institutional Animal Care and Use Committee approved the experimental protocols (IACUC# 002567 and 004399). Human peripheral blood mononuclear cells were purchased from AllCells, LLC. which obtains samples from screened healthy adult volunteer participating in an IRB or Human Subject Committtee approved donor program.

## Results

### Delipidated culture medium reduced proliferation of activated T cells

**Figure 1:**
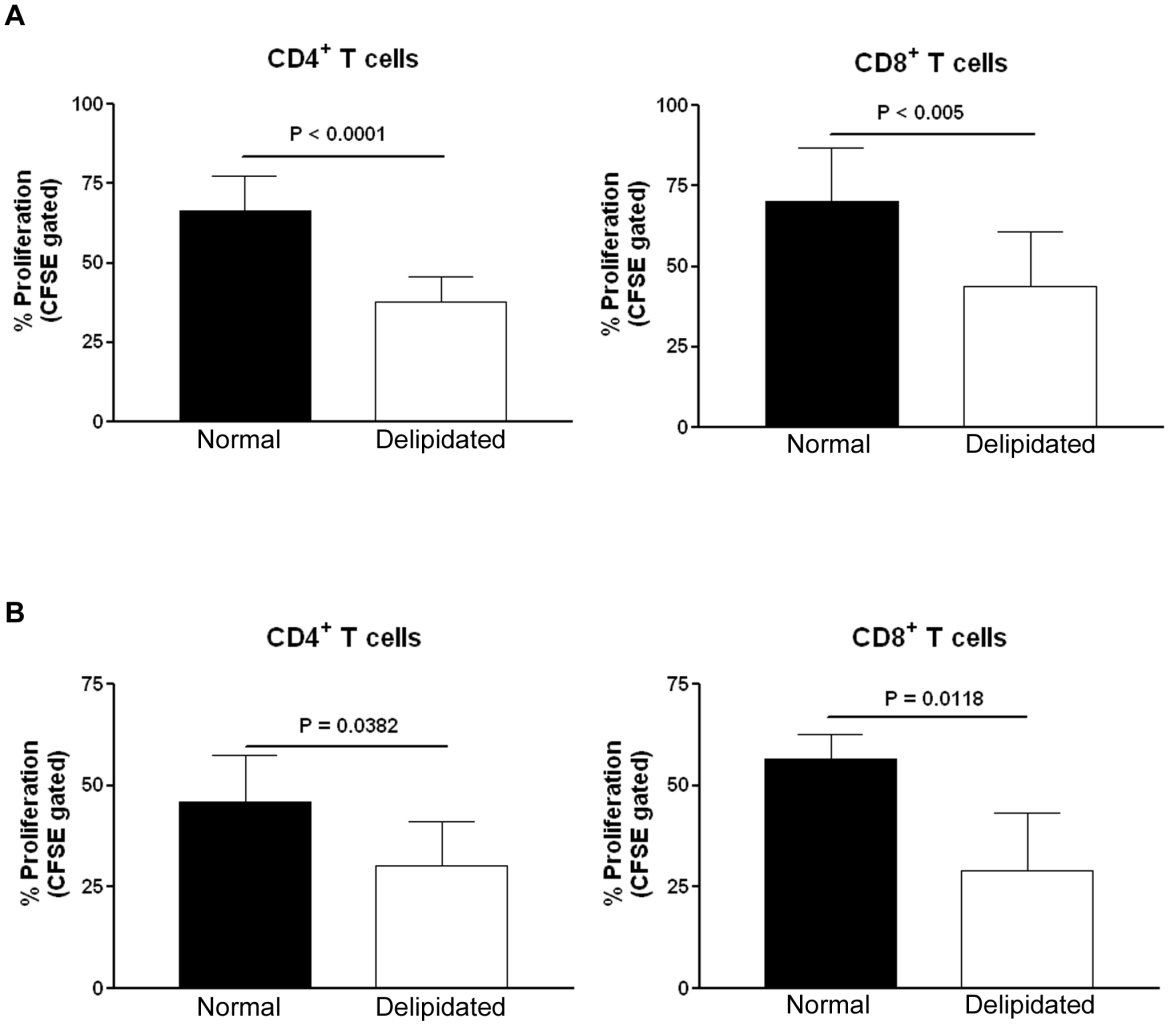
T cell proliferation in normal or delipidated medium. (A) T cells isolated from human peripheral blood were loaded with CFSE and cultured in culture medium supplemented with 10% delipidated FBS (delipidated) or 10% FBS (normal) for 4 days with CD3/CD28 activation beads. After activation, CD4^+^ and CD8^+^ T cells proliferated less when cultured in 10% delipidated FBS medium. Experiments were done in duplicates from the first sample, triplicates from the second sample and quadruplicates from the third sample using peripheral blood from 3 different human samples, hence n = 9 in each group. (B) Similarly CD4^+^ or CD8^+^ T cells isolated from naïve apoE-/- mice proliferated less when cultured in medium supplemented with 10% delipidated FBS when compared to those cultured in medium supplemented with 10% FBS. N = 6 in each group of CD4^+^ cells and n = 4 in each group of CD8^+^ cells.

We first compared the proliferative response of isolated human T cells from peripheral blood in culture medium supplemented with 10% FBS with or without lipids. After 4 days in culture medium containing 10% delipidated FBS with CD3/CD28 antibody beads to activate T cells, isolated human CD4^+^ and CD8^+^ T cells from peripheral blood proliferated much less when compared to cells in 10% FBS medium (Fig. 1A, see Fig. S1 for gating strategy).

**Figure 2:**
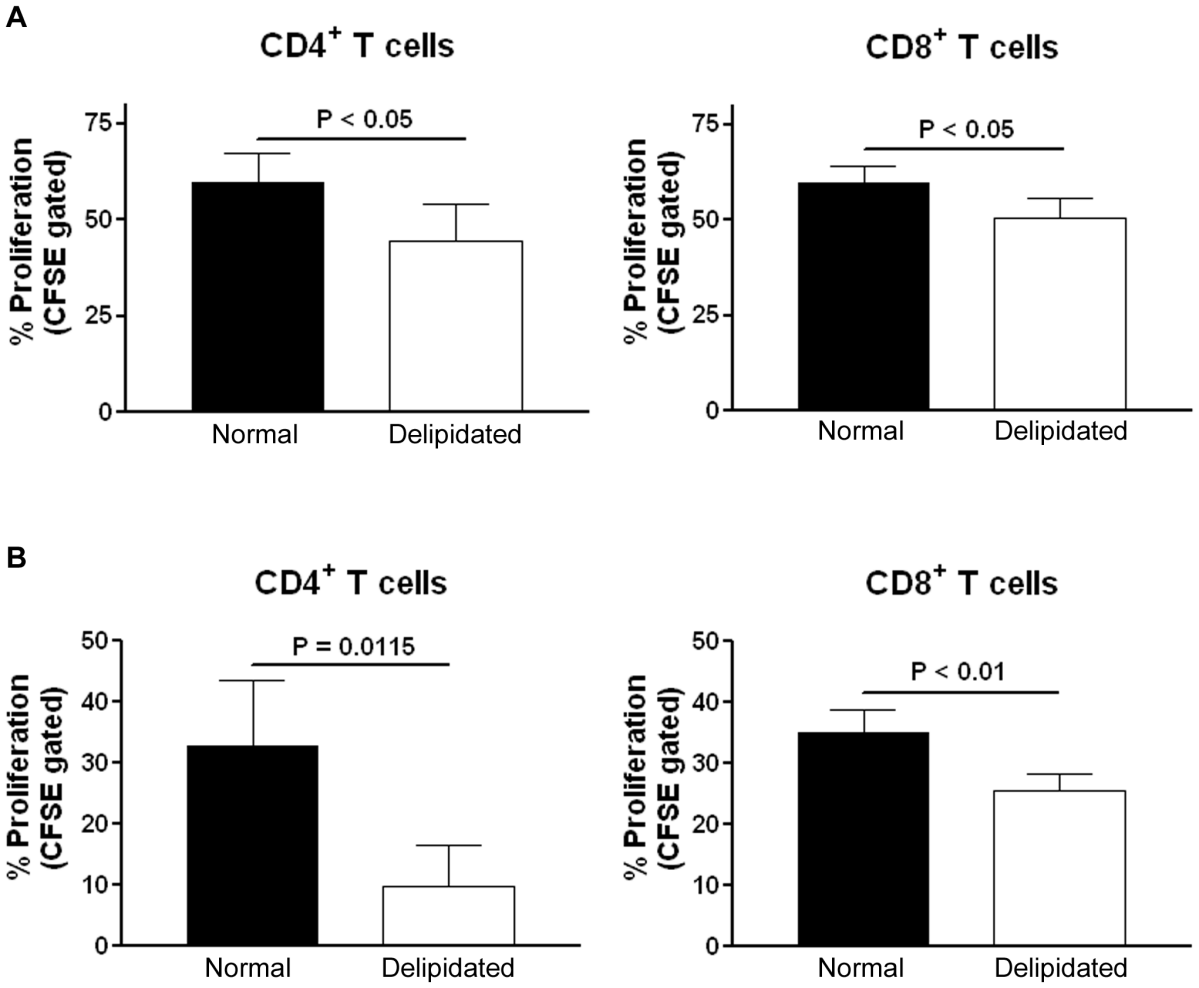
T cell proliferation in normal or delipidated meidum using serum of the same lot. (A) T cells isolated from naïve apoE-/- mice proliferated less when cultured in medium supplemented with 10% delipidated FBS when compared to those cutured in medium supplemented with 10% FBS of the same lot. N = 4. (B) T cells isolated form naïve wild type mice proliferated less when cultured in medium supplemented with 10% delipidated FBS when compared to those cutured in medium supplemented with 10% FBS of the same lot. N = 4.

We then tested if such modulation of T cell proliferation was also present in splenic T cells isolated from apoE-/- mice. Culture in 10% delipidated FBS medium also significantly reduced mouse CD4^+^ and CD8^+^ T cell proliferation stimulated by CD3/CD28 antibody beads (Fig. 1B, gating strategy was similar to that for human T cells). To eliminate the possibility that the results observed were due to differences in biochemical contents in different lots of serum, the experiment was repeated using FBS and delipidated FBS of the same lot. T cells isolated from apoE-/- mice proliferated significantly less in delipidated medium made with delipidated serum from the same lot as the normal serum (Fig. 2A). Identical experiments using T cells isolated from wild type mice showed the same results (Fig. 2B).

### Delipidated culture medium reduced cellular unesterified cholesterol content in activated T cells isolated from apoE-/- mice

**Figure 3:**
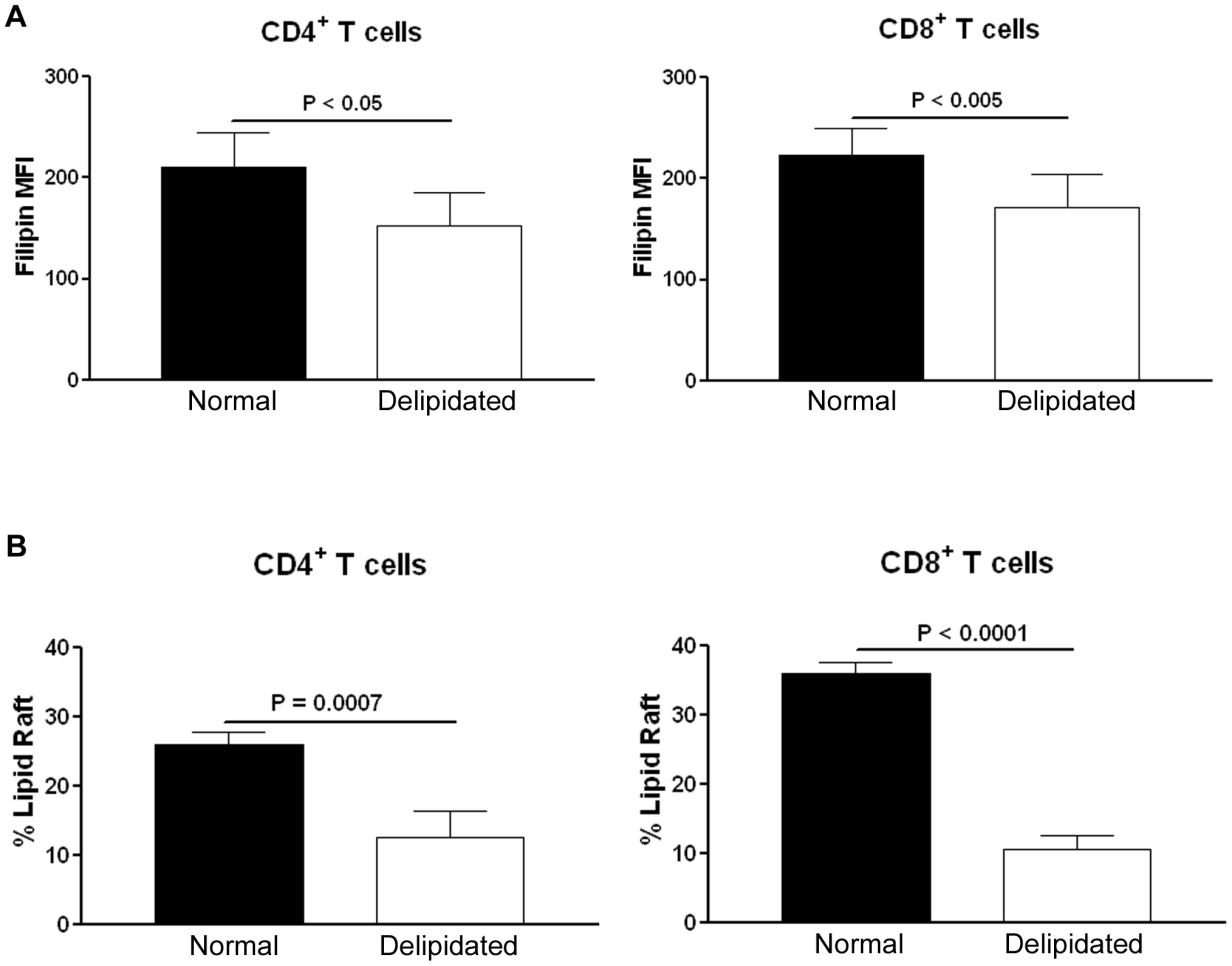
Lipid raft and cholesterol content in T cells cultured in normal or delipidated medium. T cells isolated from naïve apoE-/- mice were cultured in culture medium supplemented with 10% delipidated FBS or 10% FBS medium for 24 hours with CD3/CD28 activation beads. More unesterified cholesterol contents (A) or lipid rafts (B) were observed in activated CD4^+^ and CD8^+^ T cells cultured in 10% FBS medium when compared to T cells cultured in 10% delipidated FBS medium. Experiments were repeated 4 times using T cells from 3 mice.

T cell plasma membrane cholesterol content affects its function [18]. The reduced proliferative response of T cells in the delipidated medium suggested that the reduced response observed in low lipid milieu may be due to reduced membrane cholesterol content. We therefore tested if T cells cultured in delipidated medium would have less cellular unesterified cholesterol content using the mean fluorescent intensity (MFI) measurement of Filipin staining. CD4^+^ T cells from spleens of apoE-/- mice cultured for 24 hours in delipidated medium had significantly less Filipin MFI after stimulation with CD3/CD28 antibody beads when compared to cells cultured in normal medium (Fig. 3A, see Fig. S2 for gating strategy). Similar results were observed in CD8^+^ T cells from spleens of apoE-/- mice after stimulation with CD3/CD28 antibody beads (Fig. 3A, gating strategy was similar to that for CD4^+^ T cells).

### Delipidated culture medium reduced membrane lipid raft in activated T cells isolated from apoE-/- mice

Changes in membrane cholesterol content lead to re-organization of the lipid raft architecture affecting T cell functions [19], [20]. We analyzed the membrane lipid raft in murine T cells to further delineate if there was change in lipid raft associated with the reduced activated state of T cells in delipidated culture environment. When T cells from spleens of apoE-/- mice were cultured in normal medium, there was a significantly higher content of lipid rafts in both CD4^+^ and CD8^+^ T cells stimulated with CD3/CD28 beads (Fig. 3B, see Fig. S3 for gating strategy).

### Delipidated medium reduced phosphorylation of Zap70 in activated T cells of apoE-/- mice

**Figure 4:**
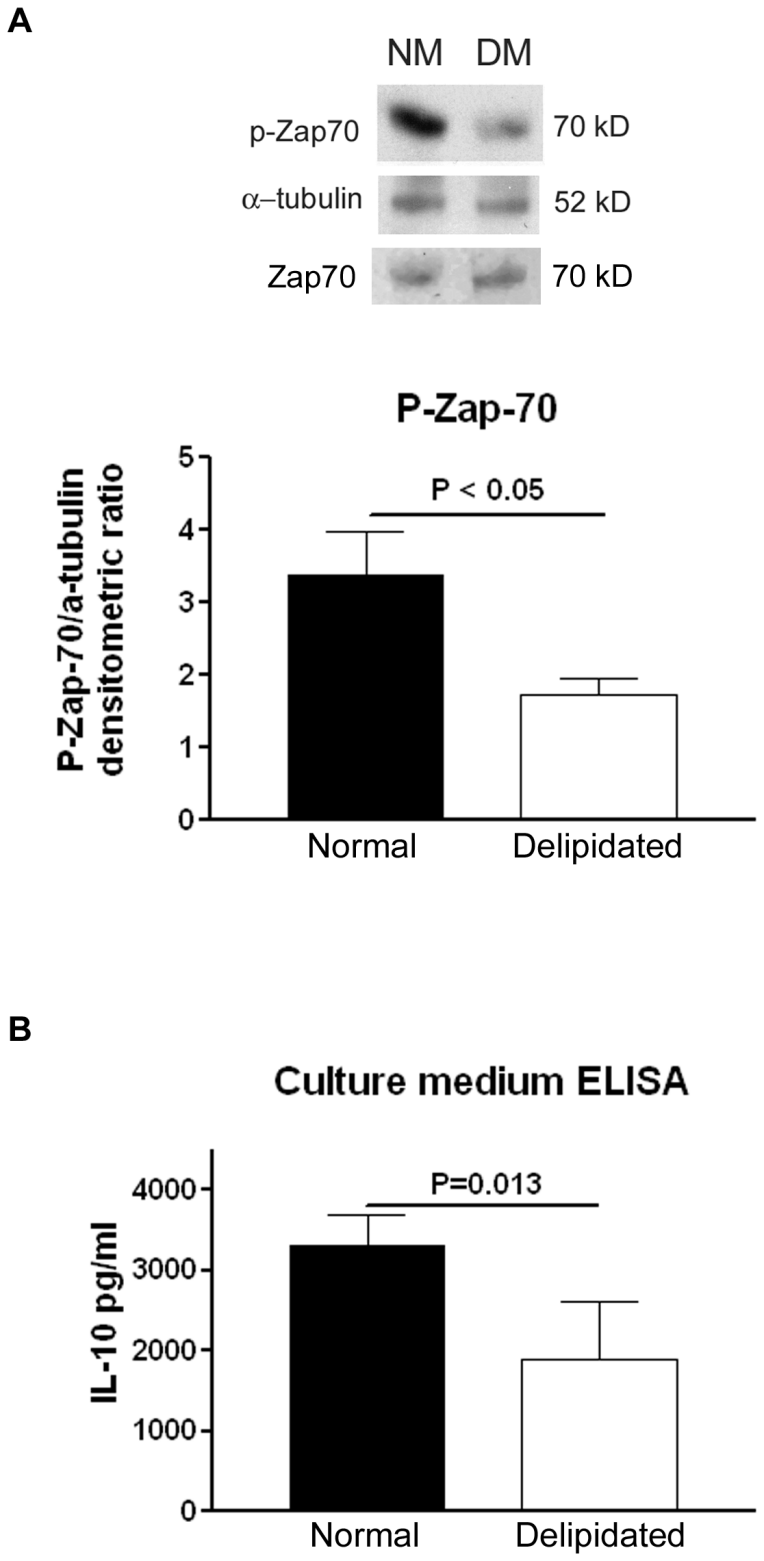
Western blot for pZap70 and IL-10 ELISA. (A) T cells isolated from naïve apoE-/- mice were cultured in 10% delipidated FBS medium or 10% FBS medium for 2 min with CD3/CD28 activation beads. Western blot analysis of the whole cell lysates revealed a higher expression of pZap-70 from T cells cultured in NM (medium supplemented with 10% FBS) when compared to that from T cells cultured in DM (medium supplemented with 10% delipidated FBS). Upper panel showed a representative Western blot and lower panel is the densitometric analysis from three experiments. N = 3 in each group. (B) IL-10 level was lower in delipidated medium of T cells after activation for 4 days with CD3/CD28 beads when compared to that in normal medium. N = 4.

The reduced proliferative response, unesterified cholesterol in plasma membrane and lipid rafts in murine T cells cultured in delipidated medium suggested that T cell signaling would also be altered. We assessed phosphorylated Zap70 expression in T cells from spleens of apoE-/- mice after stimulation with CD3/CD28 beads in normal medium or delipidated medium. There was significantly reduced pZap70 in T cells stimulated in delipidated medium (Fig. 4A).

### Delipidated medium reduced IL-10 secretion in activated T cells isolated from apoE-/- mice

A change in T cell proliferative response and signaling would likely affect cytokine secretion from T cells [21]. We hence determined the level of cytokines in culture medium collected from the 4-day T cell activation experiment from apoE-/- mice listed above. The level of IL-10 in normal medium of T cells after 4 days of activation with CD3/CD28 beads was significantly higher than that in delipidated medium (Fig. 4B). The level of IL-12 was undetectable while there was no difference in IFN-γ level between normal medium and delipidated medium after 4 days of activation (Data not shown).

### Dietary lipid lowering alters atherosclerotic plaques and T cell cytokine expression in vivo

Taking the above results together, our data suggested that reducing the lipid milieu would result in alteration of T cell function. Since hypercholesterolemia is very pronounced in atherogenic diet-fed apoE-/- mice, we used this model to test if cholesterol lowering by dietary change after prolonged consumption of atherogenic high cholesterol diet without pharmacologic intervention would alter T cell function (See Fig. S4 for diet modification strategy).

**Figure 5:**
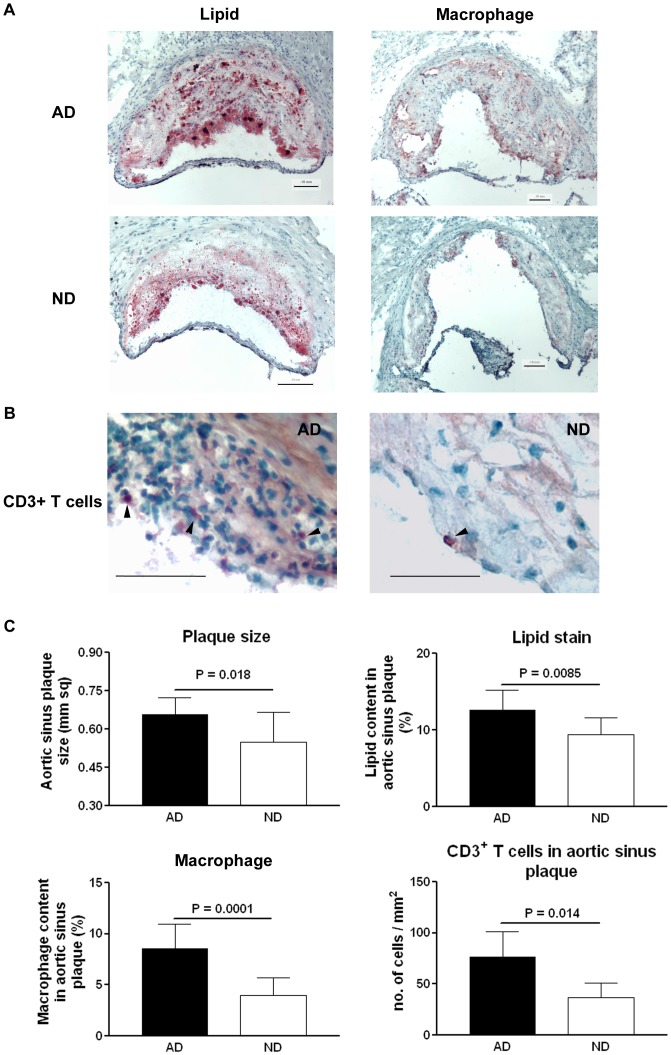
Effects of diet change on atherosclerotic plaques in aortic sinus from apoE-/- mice. (A) Representative photograph of Oil-Red-O and macrophage (MOMA-2) staining of atherosclerotic plaques in the aortic sinus of atherogenic diet (AD) and normal diet (ND) mice. Bar  = 0.1 mm. (B) Representative photograph of CD3^+^ T cell staining of atherosclerotic plaques in the aortic sinus of atherogenic diet (AD) and normal diet (ND) mice. Bar  = 0.1 mm. (C) Histomorphometric measurements of each staining are shown in bar graphs. N = 10 in each group for plaque size, lipid content and macrophage content. N = 5 in each group for CD3^+^ T cell content.

At the time of euthanasia at 29 weeks of age, ND mice had significantly lower circulating levels of total and free cholesterol when compared to those in AD mice and there was no significant difference of their body weight (Table 1). ND mice developed smaller atherosclerotic plaques in aortic sinuses when compared to those in AD mice (Fig. 5A and C). Dietary cholesterol lowering also resulted in a less inflammatory phenotype in the aortic sinus plaques from ND mice as evidenced by less lipid, macrophage and T cell immuno-reactivity in the plaques when compared to those from AD mice (Fig. 5, see Fig. S5 for negative controls of staining).

**Table 1:** Effects of diet change on apoE-/- mice.

	Body Weight (g)	Total Cholesterol (mg/dL)	Free Cholesterol (mg/dL)
AD	34.8±4.8	920±270	232±51
ND	32.4±6.0	474±85	144±19
P value	0.34	<0.0001	<0.0001

AD = Atherogenic diet; ND = Diet changed from AD to normal diet. All values are mean±SD; n = 10 in each group, t-test for statistic comparison.

**Figure 6:**
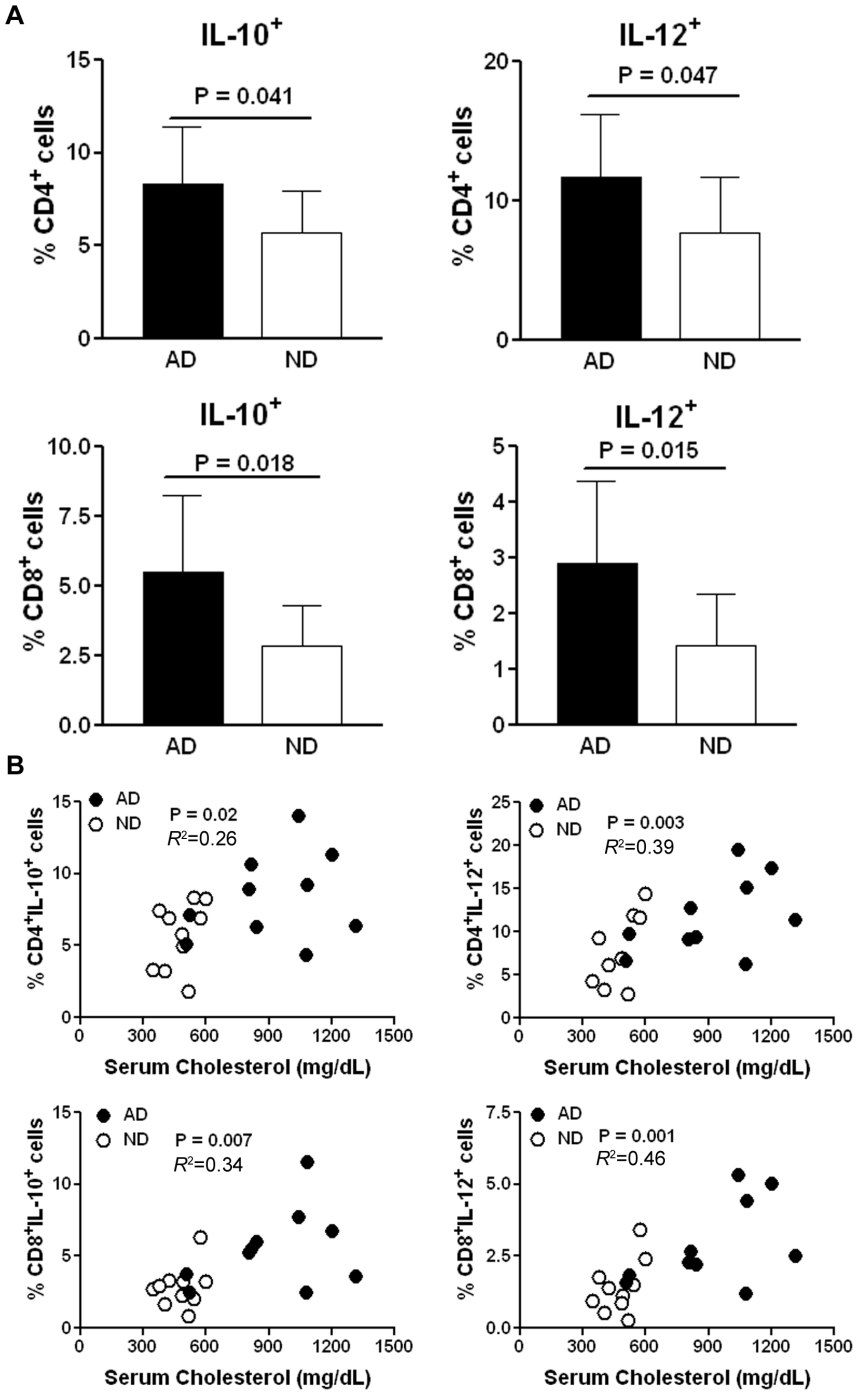
Effect of diet change on IL-10 and IL-12 in T cells and their correlation with serum cholesterol. (A) Flow cytometric analysis of splenocytes revealed that CD4^+^ and CD8^+^ T cells from AD mice express higher level of intracellular IL-10 and IL-12 when compared to those from ND mice. (B) Positive correlation was observed between serum cholesterol and the IL-10 positive or IL-12 positive in CD4^+^ or CD8^+^ T cells. N = 10 in each group. Pearson test was used for correlation.

Flow cytometric analysis of splenocytes indicated that CD4^+^ T cells from AD mice expressed significantly higher levels of intracellular IL-10 and IL-12 when compared to CD4^+^ T cells from ND mice. Similar results were observed in CD8^+^ T cells (Fig 6A, see Fig. S6 for gating strategy). There was no difference in the percentage of total splenic CD4^+^ or CD8^+^ T cells and levels of IFN-γ in CD4^+^ or CD8^+^ T cells by flow cytometric analysis (data not shown). The reduced steady state intracellular IL-10 and IL-12 levels suggested that isolated splenic CD4^+^ and CD8^+^ T cells from ND mice were less active.

The relationship between T cell status and the lipid milieu in the mice was then tested by plotting the percentage of cytokine stained T cells against serum cholesterol levels. Significant correlation was found between cholesterol levels and CD4^+^IL-10^+^ T cells (P = 0.02, *R* ^2^ = 0.26), CD4^+^IL-12^+^ T cells (P = 0.003, *R* ^2^ = 0.39), CD8^+^IL-10^+^ T cells (P = 0.007, *R* ^2^ = 0.34), and CD8^+^IL-12^+^ T cells (P = 0.001, *R* ^2^ = 0.46) (Fig. 6B).

## Discussion

In this study, we observed that (1) a reduced lipid milieu decreases cellular membrane cholesterol in T cells; (2) T cell function is altered in the reduced lipid milieu with less lipid rafts, reduced phosphorylation of Zap70, reduced IL-10 secretion and less proliferative response to polyclonal stimulation; (3) dietary cholesterol-lowering significantly reduced circulating cholesterol levels and atherosclerotic plaque sizes with a less inflammatory phenotype of plaques *in vivo*; (4) dietary cholesterol-lowering reduced cytokine expression in T cells that correlated with serum cholesterol levels. Taken together, our data show that reduced lipid milieu down-modulates T cell function.

To delineate the relationship between reduced cholesterol level and T cell function, we conducted a series of *in vitro* experiments in which T cell function was examined in culture medium with different levels of cholesterol. We observed that CD4^+^ or CD8^+^ T cells cultured in medium supplemented with 10% delipidated FBS contained reduced cellular cholesterol and membrane lipid raft, with decreased propensity for activation as shown by lower pZap70, less IL-10 secretion and less proliferation compared to T cells cultured in medium with 10% FBS. Our data thus suggested that the lipid milieu in which T cells exist affects their function with reduced activities of T cells in lipid poor environment. Lipids such as LDL or fatty acids have been known to influence T cell functions [22], [23]. Given the importance of T cells in atherosclerosis, our data suggest that the beneficial effect of cholesterol lowering by diet extends to immune function.

Both endogenous and exogenous sources of cholesterol are important in modulating lymphocyte functions. Exogenous sources of cholesterol from various fractions of lipoproteins could support lymphocyte proliferation [24]. Alteration of intracellular cholesterol metabolic pathway also modulates T cell functions. Nuclear liver X receptors (LXR) participate in cholesterol homeostasis by regulating genes in cholesterol metabolism. T cells lacking LXRβ expression proliferate more after TCR stimulation [25]. T cells from LDLR(-/-) mice genetically lacking apoA-I expression contained higher level of esterified cholesterol and were in a higher activation state in response to dietary cholesterol [10]. T cells lacking ATP-binding cassette transporter G1 (ABCG1) accumulated higher level of esterified cholesterol, membrane lipid rafts and were hyperproliferative upon TCR stimulation [11]. Our observation that a delipidated culture condition resulted in a lower level of cellular cholesterol, membrane lipid raft and decreased proliferative response to TCR stimulation in CD4^+^ or CD8^+^ T cells further strengthened the link between the lipid milieu and T cell function.

Reports that investigated the role of cholesterol in lymphocyte function in vivo have focused predominantly on lipid loading, not lipid-lowering after a period of hypercholesterolemia. For example, mice on high cholesterol chow had reduced IFN-γ, IL-4 and IL-10 secreting cells in spleen resulting in a higher Th2 to Th1 cytokine ratio and a higher circulating level of TGF-β when compared to mice on normal chow [12], [13]. Hypercholesterolemia in wild type mice caused a higher proportion of activated T cells in bone marrow and rendered splenic T cells to express higher levels of inflammatory cytokines [15]. These observations indicated that hypercholesterolemia caused a functional change in T cells, but whether such changes can be reversed by cholesterol lowering has not been tested. There is little information regarding the association of *in vivo* T cell function with dietary cholesterol lowering after a period of hyper-cholesterolemia in the published literature. Recently it was reported that hypercholesterolemia progressively decreased CD4^+^ regulatory T cells in the aorta as the mice aged and cholesterol diet continued. Dietary lowering of hypercholesterolemia prevented such a decrease of CD4^+^ regulatory T cells in the aorta. On the other hand effector CD4^+^ T cells progressively increased while on cholesterol diet but reversal of hypercholesterolemia did not reduce these cells in aortic lesions [26]. In our study we observed that dietary cholesterol lowering led to a reduced expression of intracellular IL-10 and IL-12 in CD4^+^ and CD8^+^ T cells from mice with diet switched to normal chow (ND group), suggesting T cells are less active in mice with cholesterol lowering. The correlation between serum cholesterol levels and the cytokine expression of the T cell subsets in our study also supports this notion.

Dietary and life style intervention has been known to slow the progression or promote modest regression of atherosclerotic lesions in human as demonstrated in several randomized clinical trials [27]–[29], hence such intervention becomes the first step in the management of patients with atherosclerotic cardiovascular diseases. However these clinical trials seldom address the potential mechanisms underlying the observed favorable clinical end-ponts. In pre-clinical animal studies dietary cholesterol-lowering favorably modified atherosclerotic plaques to a less inflammatory phenotype [4], [5], [30]. These pre-clinical studies provided evidence that cholesterol lowering reduces inflammation but did not address if the immune function was concomitantly modulated by dietary cholesterol lowering. Our *in vivo* observation provides experimental evidence to link inflammation reduction to a less activated state of T cells with cholesterol lowering.

The model we used allowed us to mimic a clinical condition with lipid lowering by diet and to test if such change affects T cell function. A limitation of our study is the use of atherogenic diet-fed apoE-/- mice since the serum cholesterol levels far exceed what is typical in human atherosclerotic disease. However, this limitation should not minimize the striking effect of dietary cholesterol lowering on the T cell profile after prolonged hypercholesterolemia. That the T cell profile was favorably affected in the model we used underscores an important potential link between clinical lipid management and immune function.

Cholesterol, inflammatory responses and immune activation are the major components responsible for atherosclerosis. Modulation of any of these components modifies the process of atherogenesis. Our results demonstrate that the beneficial effects of dietary lipid-lowering not only affect inflammatory responses but extend into the adaptive immune system, specifically T cell responses. The report provides a new perspective on the role of cholesterol lowering in modulating the inflammatory sequelae in atherosclerosis.

## Supporting Information

Figure S1 **Gating strategy for CD4^+^ or CD8^+^ proliferating CFSE labeled total T cells isolated from human peripheral blood after 4 days of culturing in 10% delipidated FBS medium or 10% FBS medium with CD3/CD28 activation beads.** Cells were first gated on forward and side scatter, then CD4^+^ or CD8^+^ T cells were selected and a fixed gate was applied to determine the percentage of proliferation on all samples based on the shifting of CFSE to the left. Murine T cells were gated using the same method.
(PDF)

Figure S2 **Gating strategy for the quantification of unesterified cholesterol in CD4^+^ or CD8^+^ in T cells from naïve apoE(-/-) mice that were cultured for 24 hours in 10% FBS medium or 10% delipidated FBS medium with CD3/CD28 activation beads.** Cells were first gated on forward and side scatter, then APC-efluor 780 labeled CD4^+^ were selected and the amount of unesterified cholesterol was quantitated by mean fluorescent intensity (MFI) measurement of Filipin staining. APC labeled CD8^+^ cells were gated using the same method.
(PDF)

Figure S3 **Gating strategy for percentage of lipid raft on CD4^+^ or CD8^+^ in T cells from naïve apoE(-/-) mice that were cultured for 24 hours in 10% FBS medium or 10% delipidated FBS medium with CD3/CD28 activation beads.** Cells were first gated on forward and side scatter, then PE labeled CD4^+^ were selected and a fixed gate was applied to determine the percentage of lipid raft on all samples based on the increased in intensity of Alexa Fluor 488 labeled Cholera Toxin Subunit B. PerCP-eFluor 710 labeled CD8^+^ cells were gated using the same method.
(PDF)

Figure S4 **In vivo study experimental design.**
(PDF)

Figure S5 **Negative controls of immunostaining.**
(PDF)

Figure S6 **Gating strategy for IL10^+^ or IL12^+^ on CD4^+^ or CD8^+^ cells.** Total splenocytes from AD mice or ND mice were first gated on FITC labeled CD4^+^ or PE labeled CD8^+^ cells and then APC labeled IL10^+^ or PerCP-Cy5.5 labeled IL12^+^ cells that were also positive for CD4 or CD8 were selected in the upper right region as shown.
(PDF)
