# Supplementary figures and images for: Cholesterol Lowering Modulates T Cell Function In Vivo and In Vitro
Source: PLoS One. 2014 Mar 19;9(3):e92095. doi: 10.1371/journal.pone.0092095 (PMC3960213; doi:10.1371/journal.pone.0092095)

**Fig. S1**

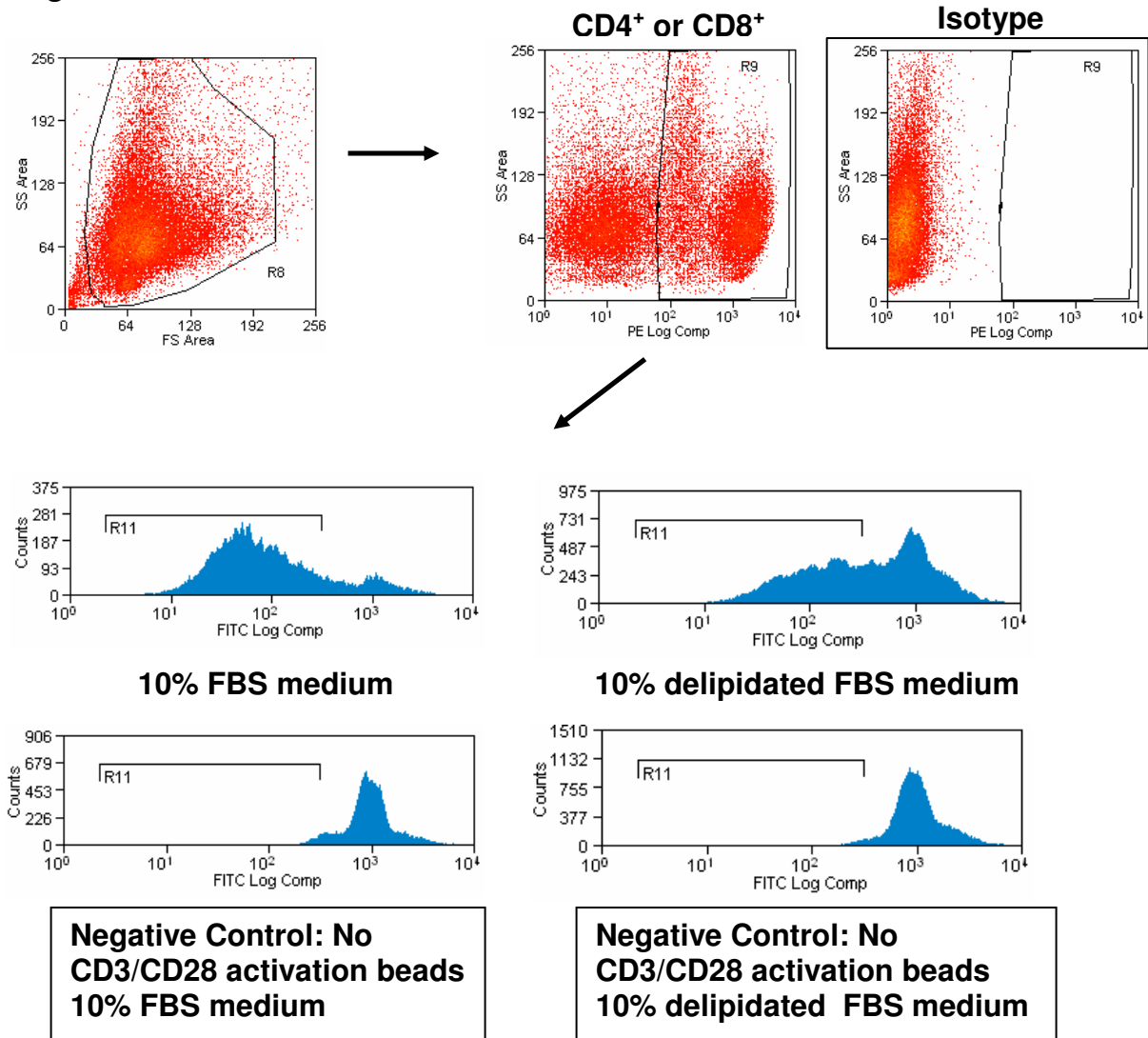

Supplement: Figure S1 — Gating strategy for CD4+ or CD8+ proliferating CFSE labeled total T cells isolated from human peripheral blood after 4 days of culturing in 10% delipidated FBS medium or 10% FBS medium with CD3/CD28 activation beads. Cells were first gated on forward and side scatter, then CD4+ or CD8+ T cells were selected and a fixed gate was applied to determine the percentage of proliferation on all samples based on the shifting of CFSE to the left. Murine T cells were gated using the same method. (PDF) [file pone.0092095.s001.pdf]

**Fig. S2**

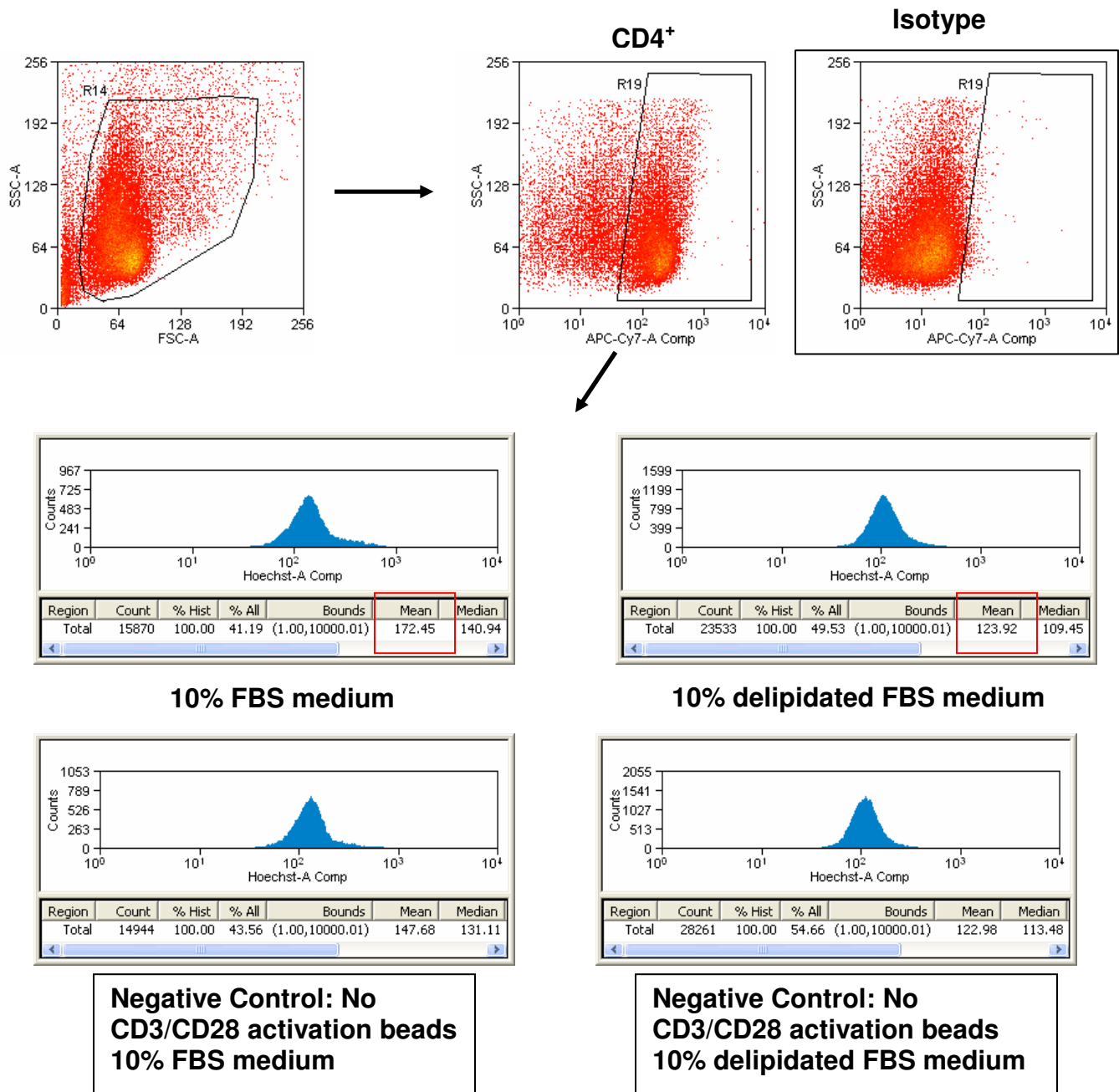

Supplement: Figure S2 — Gating strategy for the quantification of unesterified cholesterol in CD4+ or CD8+ in T cells from naïve apoE(-/-) mice that were cultured for 24 hours in 10% FBS medium or 10% delipidated FBS medium with CD3/CD28 activation beads. Cells were first gated on forward and side scatter, then APC-efluor 780 labeled CD4+ were selected and the amount of unesterified cholesterol was quantitated by mean fluorescent intensity (MFI) measurement of Filipin staining. APC labeled CD8+ cells were gated using the same method. (PDF) [file pone.0092095.s002.pdf]

**Fig. S3**

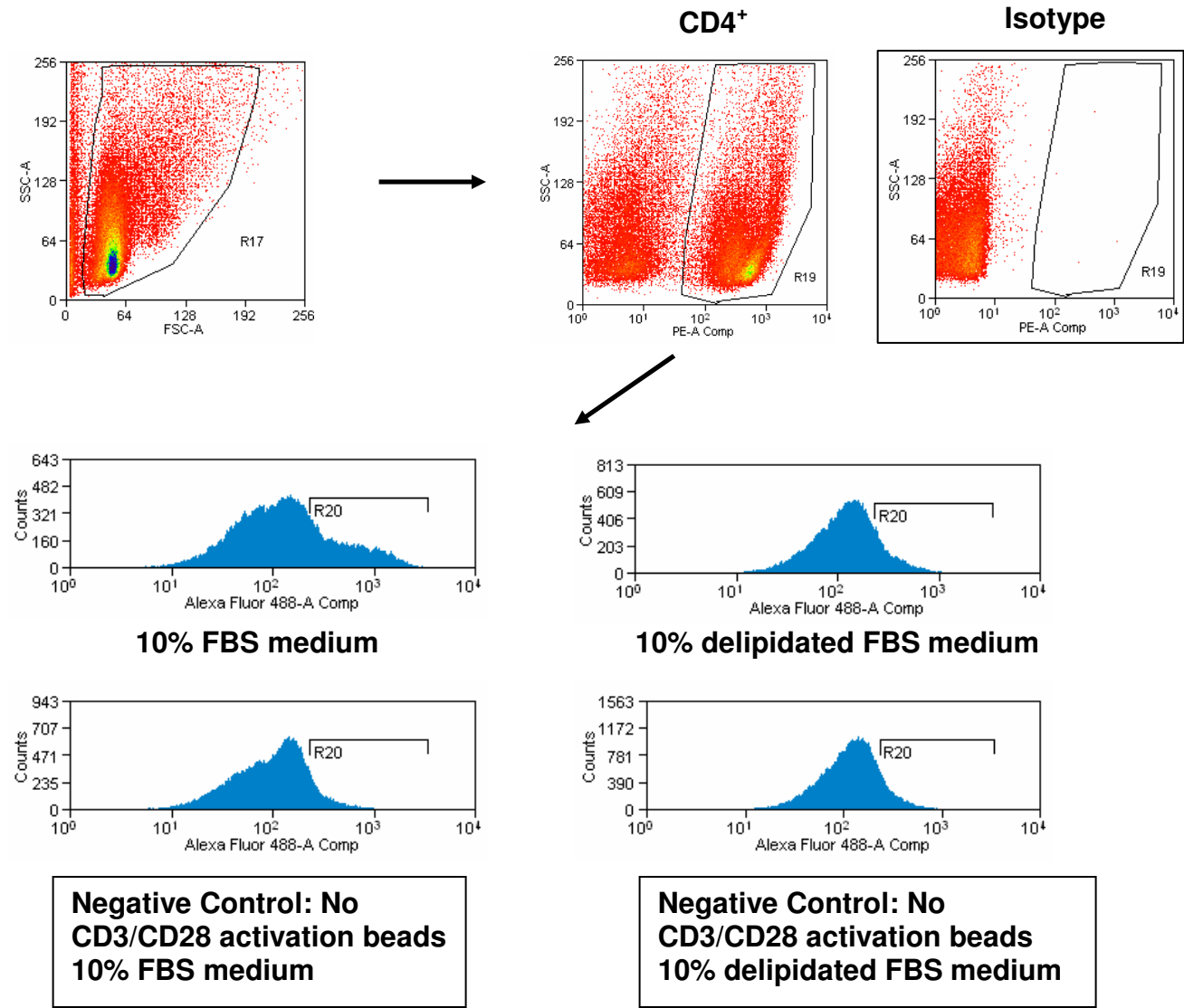

Supplement: Figure S3 — Gating strategy for percentage of lipid raft on CD4+ or CD8+ in T cells from naïve apoE(-/-) mice that were cultured for 24 hours in 10% FBS medium or 10% delipidated FBS medium with CD3/CD28 activation beads. Cells were first gated on forward and side scatter, then PE labeled CD4+ were selected and a fixed gate was applied to determine the percentage of lipid raft on all samples based on the increased in intensity of Alexa Fluor 488 labeled Cholera Toxin Subunit B. PerCP-eFluor 710 labeled CD8+ cells were gated using the same method. (PDF) [file pone.0092095.s003.pdf]

**Fig. S4 In vivo study experimental design.**

- *Subject: Male apoE (-/-) mice*

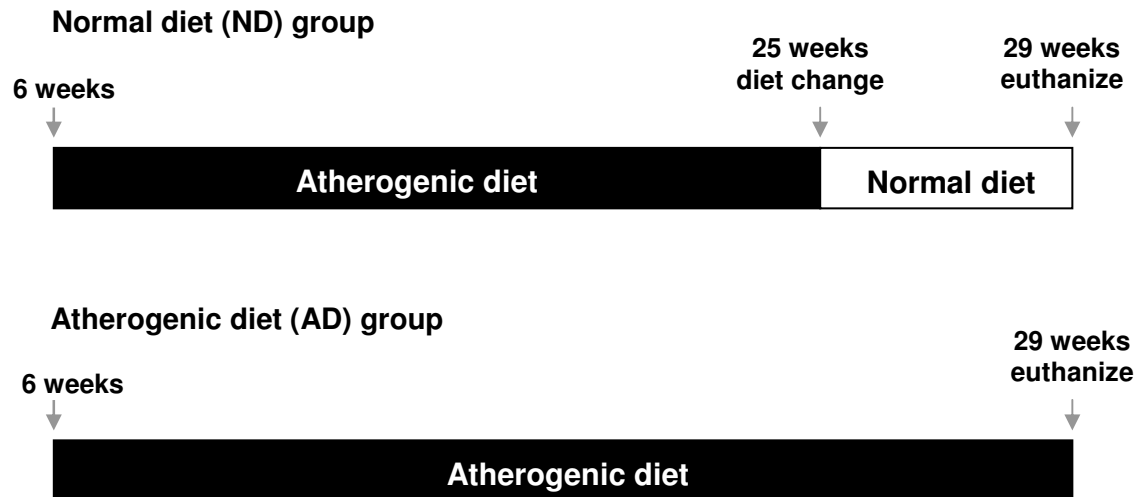

Supplement: Figure S4 — In vivo study experimental design. (PDF) [file pone.0092095.s004.pdf]

**Fig. S6**

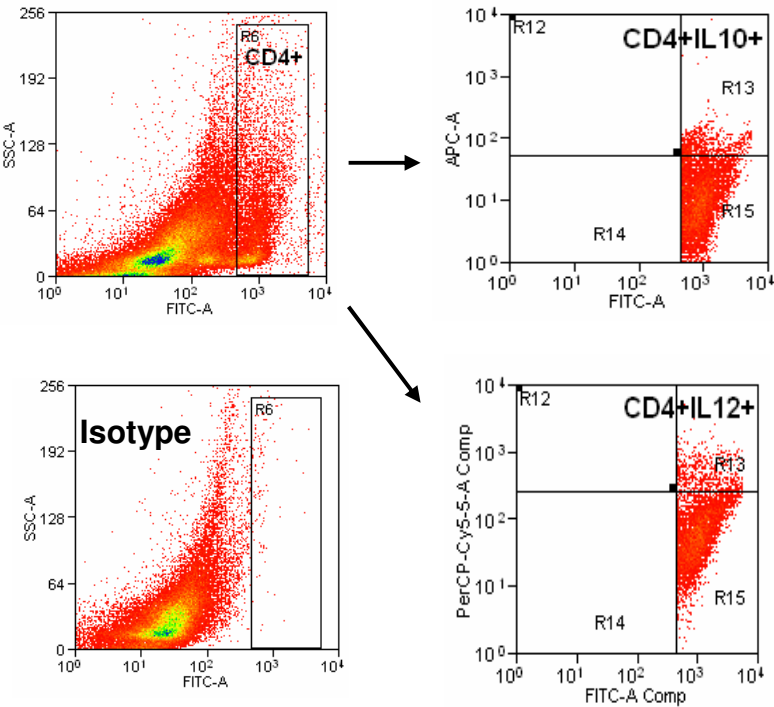

Supplement: Figure S6 — Gating strategy for IL10+ or IL12+ on CD4+ or CD8+ cells. Total splenocytes from AD mice or ND mice were first gated on FITC labeled CD4+ or PE labeled CD8+ cells and then APC labeled IL10+ or PerCP-Cy5.5 labeled IL12+ cells that were also positive for CD4 or CD8 were selected in the upper right region as shown. (PDF) [file pone.0092095.s006.pdf]
