# Supplementary material for: Cholesterol Lowering Modulates T Cell Function In Vivo and In Vitro
Source: PLoS One. 2014 Mar 19;9(3):e92095. doi: 10.1371/journal.pone.0092095 (PMC3960213; doi:10.1371/journal.pone.0092095)

**Fig. S5**

Omission of MOMA-2 primary antibody as negative control for macrophage immune-reactivity. Bar = 0.1 mm at 10X magnification.

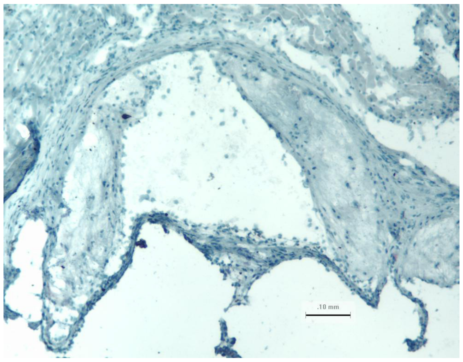

Omission of CD3 antibody as negative controls for CD3 T cell immune-reactivity. Bar = 0.1 mm at 40X magnification.

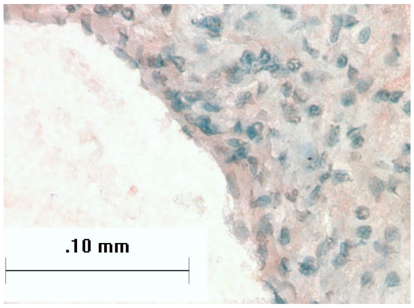

Supplement: Figure S5 — Negative controls of immunostaining. (PDF) [file pone.0092095.s005.pdf]
